# Advantages of Lateral Flow Assays Based on Fluorescent Submicrospheres and Quantum Dots for *Clostridium difficile* Toxin B Detection

**DOI:** 10.3390/toxins12110722

**Published:** 2020-11-19

**Authors:** Haonan Qi, Qiuli Sun, Yi Ma, Peidian Wu, Jufang Wang

**Affiliations:** 1 School of Biology and Biological Engineering, South China University of Technology, Guangzhou 510006, China; haonanqi2020@163.com (H.Q.); sunqiuli2013@163.com (Q.S.); bimayikobe@scut.edu.cn (Y.M.); 2 National & Local United Engineering Lab of Rapid Diagnostic Test, Guangzhou Wondfo Biotech Co., Ltd., Guangzhou 510663, China; dpwu@wondfo.com.cn

**Keywords:** lateral flow assay, fluorescent submicrospheres, quantum dot nanobead, toxin B

## Abstract

*Clostridium difficile* colitis is caused by a cytotoxin produced by the anaerobic bacteria *C. difficile* in the epithelial cells of the large intestine, particularly *C. difficile* toxin B (TcdB). However, the sensitivity of currently utilized *C. difficile* endotoxin determination methods has been called into question, and, therefore, more accurate and convenient detection methods are needed. Our study is the first to systematically compare fluorescent submicrosphere-based and quantum-dot nanobead-based lateral fluidity measurement methods (FMs-LFA and QDNBs-LFA) with toxin B quantification in fecal samples via sandwich analysis. The limits of detection (LOD) of FMs-LFA and QDNBs-LFA in the fecal samples were 0.483 and 0.297 ng/mL, respectively. TcdB analyses of the fecal samples indicated that the results of QDNBs-LFA and FMs-LFA were consistent with those of a commercial enzyme-linked immunosorbent assay (ELISA) test kit. The sensitivity of QDNBs-LFA was highly correlated with clinical diagnoses. Therefore, quantum dot nanobeads (QDNBs) are deemed highly suitable for lateral fluidity analyses, which would facilitate the implementation of portable and rapid on-the-spot applications, such as food hygiene and safety tests and onsite medical testing.

## 1. Introduction

*Clostridium difficile* (*C. difficile*), a strictly anaerobic, spore-forming, Gram-positive bacillus, is considered the main pathogen responsible for the widespread occurrence of antibiotic-associated diarrhea in hospitals. *C. difficile* infection (CDI) is the primary factor contributing to diarrhea in medical facilities, often affecting the most susceptible antibiotic-treated populations. This pathogen is also responsible for approximately 2% of community-acquired diarrhea cases [1,2]. Only the toxigenic strains that generate toxins A and B have been linked to disease, of which TcdB is considered the main mechanism of CDI onset. In the last decade, issues from *C. difficile*-induced diarrhea hospitalizations and community-acquired CDI have increased worldwide, which can be attributed to the spread of highly virulent strains [3,4,5].

In order to allow patients to receive proper treatment and to avoid the spread of disease, accurate and rapid CDI testing is particularly important. Inpatients that have been infected with toxigenic *C. difficile* strains may remain free of symptoms [6]. Therefore, identifying toxin-producing strains in the fecal samples of symptomatic patients does not necessarily determine the cause of diarrhea. Instead, the amount of *C. difficile* toxin in feces might be more descriptive of CDI severity than identifying toxigenic isolates (toxigenic culture (TC)) from bacterial cultures. Cytotoxicity measurement (CCTA) is considered the “gold standard” for *C. difficile* toxin detection (mostly toxin B) in excreta [7]. However, this approach requires 24 to 48 h to render results. The nucleic acid amplification tests (NAATs) also have limitations, namely, high cost and some interpretation difficulties [5]. NAATs were introduced in 2009. These tests are based on either a PCR method or isothermal amplification. Alternatively, commercial enzyme immunoassays (EIAs) and lateral flow assay methods for assessing toxins A and B are also commonly used. However, these tests exhibit significant fundamental differences [8,9].

Test strips provide a quick and easy method that can be performed onsite and in a single step. Additionally, this approach is also a customer-friendly and low-cost alternative for toxin B quantification, which facilitates its potential onsite implementation. Additionally, the widespread implementation of organic fluorescence is by photobleaching effects, which largely decrease assay sensitivity. Lateral flow immunochromatography (LFA) methods for different markers [10] such as ICA (GNP-LFA) using gold nanoparticles (GNP) [10,11,12], fluorescent-microsphere-derived ICA (F-ICA), and time-resolved Eu/Tb(III) nanoparticles ICA (TRF-ICA) [13,14] have been designed for the determination of harmful substances in food. Moreover, these semiquantitative or quantitative analytical methods are simple, easy, fast, and require only visual inspection.

Recently, quantum dots (QDs) have received increasing attention and are reportedly among the best potential candidates for the development of novel detection methods [15]. In comparison to organic chemical fluorescent groups, quantum dot technology has a high quantum yield, excellent optical reliability, wide absorption cross-sections, adjustable specification of fluorescent transmission, narrow spectral linewidth, and excellent antibleaching reliability. However, it is not clear whether QDs alone would be less sensitive due to long-term organic chemistry asymmetry and colloidal solutions in the natural physiological environment after the entire phase of the migration process [16]. In order to address this problem, inclusion or encapsulated magnetic beads have been proposed as signal indicators to obtain excellent organic chemistry and colloidal solution reliability. For example, Li et al. used quantum dot nanobeads (QDNBs) as a probe to identify specific prostate antigens. They produced a test strip and found that its limit of detection (LOD) was better (i.e., 12 times lower) than that of QDs alone [17].

In order to address matrix effects, a nonspecific anti-toxin B monoclonal antibody, with a high aspect ratio, was developed herein. Based on the physical, chemical, and electron optic characteristics of QDNBs and fluorescent submicrospheres (FMs), our study developed and designed two novel side LFAs based on QDNBs and FMs to quickly and accurately test for TcdB in stool samples. Spiked test specimens were used to validate quantification characteristics, including LOD, linear category, utilization rate, and relative standard deviation (RSD). The results of the test strip and the ELISA test kit were then further compared.

## 2. Results

### 2.1. Methodological Principles

**Figure 1:**
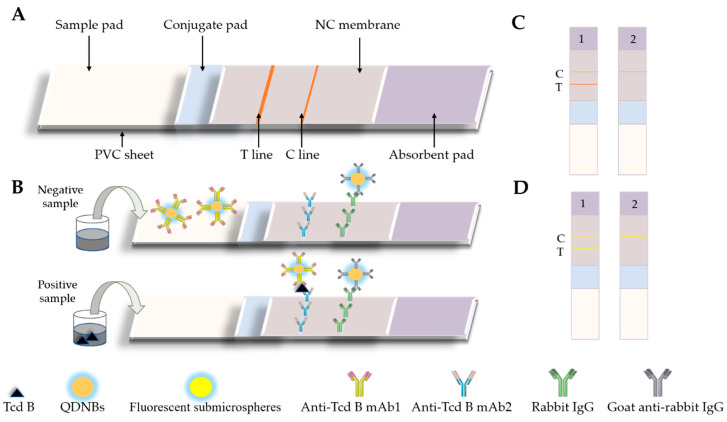
Schematic of the quantum-dot nanobead-based lateral fluidity assessment (QDNBs-LFA) and fluorescent submicrosphere-based lateral fluidity assessment (FMs-LFA) strips. (**A**,**B**) Structure of the LFA strip and test procedure for the LFAs. (**C**) QDNBs-LFA strips:—1: positive result; 2: negative result. (**D**) FMs-LFA strips—1: positive result; 2: negative result.

Figure 1A illustrates a diagram of the LFA framework. The test strip was fabricated with a test sample pad, an absorbent pad, a nitrocellulose (NC) membrane, and a conjugate pad. A 50-mM borate solution (pH 7.4), which contained sodium azide (0.05%), Tween-20 (0.5%), and BSA (1%), was used to resolve the test pad, followed by drying for 2 h at 60 °C. Rabbit IgG (0.6 mg/mL) was assigned to the NC membrane, as well as the connection and comparison lines, and then dried at 37 °C for 12 h. In Figure 1B, the labeled probe was incubated with 100 μL of PBS or excrement sample in an ELISA well at room temperature for five minutes. The compound was then pipetted into the test well of the test strip. Once the liquid sample was transferred onto the test strip, the antigen was fused with the labeled mAb probe. The labeled rabbit IgG could then be observed with Line C. For comparative measurements, the labeled mAb probe was randomly transferred to the NC membrane and was observed at the C line. As showned in Figure 1C,D, in 12 to 15 min, the LFA was analyzed with specialized reading software. The optical signal results were then obtained with a portable reader. Qualitative toxins analyses were performed using the fluorescent compressive strength of the recorded T and C lines. The inherent and background heterogeneity was determined from the T/C ratio.

### 2.2. Characterization of Label Sizes and Transmission of Labeled pAb Probe 

**Figure 2:**
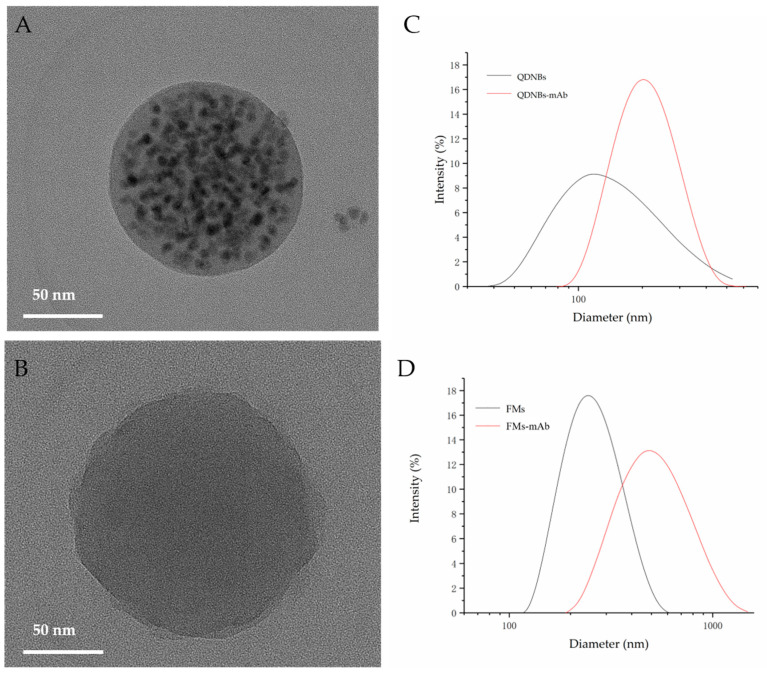
Size characterization of QDNBs and fluorescent submicrospheres. (**A**,**B**) TEM image of QDNBs and fluorescent submicrospheres. (**C**,**D**) Dynamic light scattering spectrum of QDNBs and fluorescent submicrospheres.

According to transmission electron microscopy (TEM; Figure 2A,B), QDNBs and FMs have relatively well-proportioned specifications, with average diameters of 90 and 200 nm, respectively. After conjugating anti-TcdB mAb to the surface of the nanoparticles, the mean hydrodynamic diameters of QDNBs and FMs (Figure 2C,D) were 144.8, 255 to 196.8, and 712.4 nm, respectively. The polydispersity index (PDI) values of QDNBs and FMs are 0.091 and 0.032, respectively. After the particles and the antigen were fused, these PDI values were slightly increased to 0.332 and 0.380. PDI values are used as symmetry indicators. Specifically, aqueous solutions labeled with lower PDI values have stronger symmetry than those with a higher PDI value, in addition to a higher antigen usage rate and higher sensitivity [18]. This indicates that the anti-TcdB mAb has been successfully coupled onto the two labeled surface layers. A particle size analyzer was employed to characterize the labels and probes, and the QDNBs and QDNBs–mAb probes showed relatively low PDI values, which provide further improved optical signals.

### 2.3. Experimental Parameter Optimization

**Figure 3:**
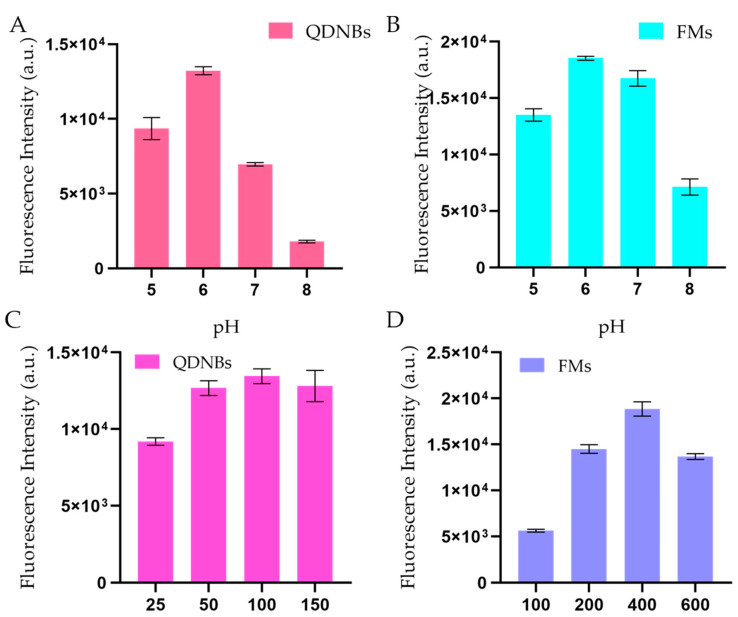
(**A**) Optimization of coupling pH (pH 5, 6, 7, and 8) between QDNBs and anti-*C. difficile* toxin B (TcdB) mAb. (**B**) Optimization of coupling pH (pH 5, 6, 7, and 8) between FMs and anti-TcdB mAb. (**C**) Optimization of QDNBs-conjugated anti-TcdB mAb concentration. (**D**) Optimization of FMs-conjugated anti-TcdB mAb concentration. The concentration of the Tcd-B-spiked sample was 50 ng/mL.

Two mAbs (C6, E6) specific to different toxin epitopes were purified in our lab [19]. Given that the antibody purity could impact the specificity and sensitivity of the assay, the antibodies were analyzed via SDS-PAGE electrophoresis and were found to be >85% pure. These two mAbs were used to capture and detect antibodies. The affinity of the C6 mAbs is 0.303 nM, E6 is 1.03 nM. It is recommended that *K_D_* values less than 14.0 nM are high-affinity antibodies; hence, both the captured and labeled antibodies showed a high affinity for TcdB detection [20]. Immunochromatographic analysis strips represent a quick quantification and detection approach but are limited by the mAb characteristics, the aqueous solution of the test product, and the immune reaction time. Moreover, this approach requires increasing the labeling pH to produce LFAs, as labeling pH compromises antigen specificity and the efficiency of the coupling reaction [21]. Fluorescent probes (QDNBs and FMs) can be prepared through the covalent coupling reaction between the carboxyl group of fluorescent nanoparticles in the EDC·HCl and the hydroxyl group of anti-TcdB mAb. EDC is most active at a pH range from 4.5 to 7.2. As shown in Figure 3A,B, the T-line-receiving sensitivity changes slightly as the pH increases, and it has the strongest compressive strength at pH 6.0. The concentration value of anti-TcdB mAb is also the primary condition for the coupling reaction with the label. As shown in Figure 3C,D, the coupling reaction rate of labeled mAb gradually increases with increasing anti-TcdB mAb concentration, and its strongest compressive strength is achieved at 100 and 400 μg/mL.

**Figure 4:**
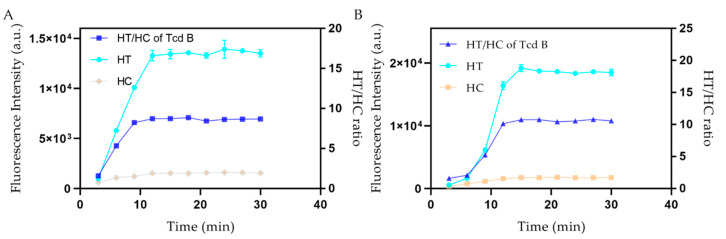
Immunoreaction dynamics of the (**A**) QDNBs-LFA and (**B**) FMs-LFA. It can be observed T and C increased further with increased reaction time, and the T/C ratio remained stable after ten minutes, demonstrating that during the entire process of fluidity transfer along the nitrocellulose (NC) membrane, the immune response can be thoroughly carried out within 12 and 15 min. The TcdB-spiked sample was 50 ng/mL.

Additionally, the effect of sample volume was also assessed. Our study determined that the labeled mAb could not interact with the antigen body during the lateral fluidity period at sample volumes below 80 μL. In the case of sample volumes larger than 100 μL, ideal measurement characteristics and great luminosity could be observed for the analysis strips. Many fluorescent probes can harm the fluorescent response of immunochromatographic analysis strips because their compressive strength depends on the quantity of probes adhered to the T line. Relatively high TcdB concentrations cannot be quantified with insufficient monoclonal antibodies. This may be because the antibody on the T network cannot fully capture the endotoxin molecules, resulting in a narrow dynamic range. However, excessive labeled mAb concentrations would reduce sensitivity due to the high background noise caused by excessive fluorescent light. In order to increase the labeled mAb concentrations, the prepared mAb label was mixed with various concentration value ratios to conduct the TcdB test. Therefore, our study determined that there were 10 µL of QDNBs–mAb present in the 100 µL sample volume, whereas the amount of FMs–mAb used in the 100 µL volume of the test sample was 15 µL. In order to estimate the reaction speed, measurements were carried out under different immune response delay times. Figure 4 illustrates the relationship between each parameter, including T, C, T/C ratio, and immune response time. T and C increased further with increased reaction time, and the T/C ratio remained stable after ten minutes, indicating that the immune response can be thoroughly carried out within 12 and 15 min during the entire process of fluidity transfer along the NC membrane. Therefore, it is best to include a 12-to-15-min interval in the reaction rate.

### 2.4. Evaluation of Immunochromatographic Strips

**Figure 5:**
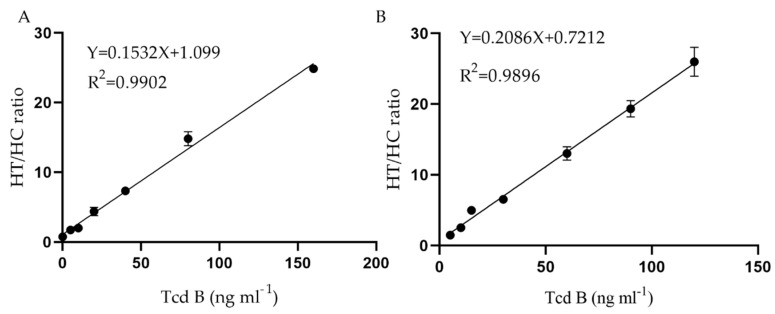
The standard calibration curve for TcdB concentrations was obtained from the HT/HC ratio. (**A**) QDNBs-LFA, (**B**) FMs-LFA. The data represent the average of three replicates.

As shown in Figure 1A, LFA was applied for quantitative analysis of TcdB according to the label. This procedure is based on the basic sandwich immunoassay. The test product was mixed with the functionalized label (QDNBs–mAb or FMs–mAb) and transferred to the digestion and absorption pad according to capillary action on the test product pad. Upon reaching the test connection line, the immune complexes were captured by the corresponding antigens on the NC membrane, thereby producing FMs(QDNBs)–antibody–antigen–antibody complexes. Throughout the process, the functional mark is fixed on the detection line, and the unnecessary mark is moved along the membrane to the digestion and absorption pad. The fluorescent data signal is accurately measured according to the fluorescent strip reader. Under normal circumstances, the concentration of antigens in the test particle is directly proportional to HT (T line signal) and its HT/HC ratio (i.e., T line to C line ratio). However, when only HT is used, it is generally difficult to carry out qualitative analysis on the resulting data signals. The original heteroskedasticity of the side stream test strips will adversely affect the credibility and accuracy of the test cultivation medium. Therefore, our study used the HT/HC ratio to carry out accurate measurements. It is worth noting that all previous studies have coated the NC membrane with goat anti-rabbit IgGs to generate a contrast system, which is different from the general lateral flow immunity data analysis system. Compared with traditional methods, labeled rabbit IgG-goat and anti-rabbit IgG nitric oxide synthase showed a more stable internal control data signal [22]. Based on the peak compressive strength ratio of the HT/HC and TcdB concentration values of the scanner, a calibration curve was plotted, where HT and HC represent the T and C lines, respectively, in the presence and absence of antigen. After repeating the test five times, 10 blank samples were used to calculate the LOD as the average value according to the calibration curve. As shown in Figure 5A, the proposed LFA sealant strips based on QDNBs mainly exhibited linearity at 0.2–160 ng/mL; the LOD in the buffer solution was 0.297 ng/mL. The explicit correction equation was Y = 0.1532X + 1.099, with a reliable correlation coefficient (R^2^ = 0.9902). As shown in Figure 5B, the proposed LFA’s miscellaneous bands, based on FMs, mainly exhibited linearity at 0.4–150 ng/mL; the LOD in the buffer solution was 0.483 ng/mL. The explicit correction equation was Y = 0.2086X + 0.7212, with a reliable correlation coefficient (R^2^ = 0.9896).

**Figure 6:**
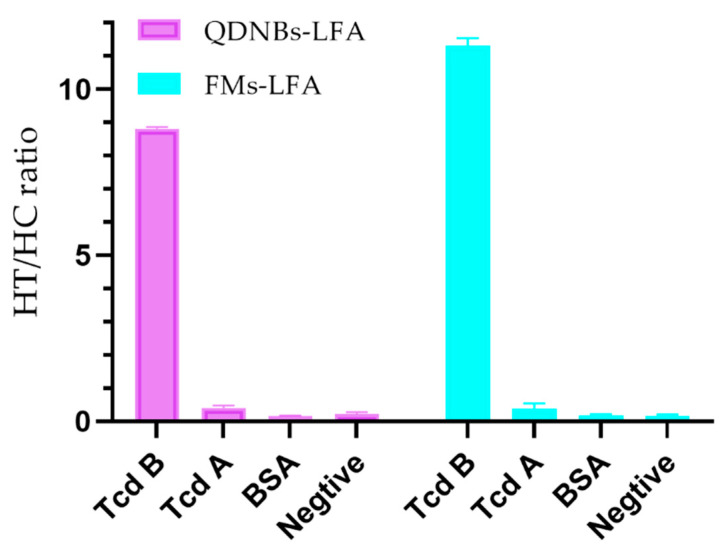
Specificity analysis of QDNBs-LFA and FMs-LFA. The concentration of *C. difficile* toxin A (TcdA) was 500 ng/mL. The data represent the average of three replicates.

In order to estimate the nonspecificity of LFA according to the labels, the cross-reflexivity between TcdB and TcdA and BSA was evaluated based on a fixed immobility value of 50 ng/mL (in triplicate). In Figure 6, the TcdA HT/HC ratio of the two LFAs was found to be as low as the TcdB negative control sample.

The utilization rate of the test sample for research purposes is based on the simulation of a series of TcdB concentration values. Table 1 summarizes that the utilization rates of FMs and QDNBs, which are 92.6% to 107.3% and 99.58% to 112.5%, respectively. As a result, it was found that all the utilization data complied with the national guidelines for immunochromatographic analysis strips.

Intra-assay and interassay specificity were evaluated based on repeated trials. Three spiked TcdB extracts, with concentration values of 20, 40, and 80 ng/mL, were introduced to ensure the reproducibility between and within batches (Table 2). This confirms why QDNBs and FMs are commonly used for quantitative TcdB analysis.

**Figure 7:**
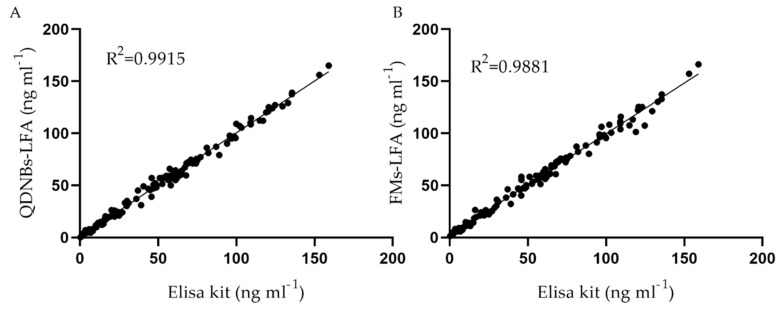
Correlation between the results from ELISA and the two LFAs in 120 stool samples with different TcdB concentrations. The linear regression equations for QDNBs (**A**) and FMs (**B**) were Y = 0.9960X + 0.8703 (R^2^ = 0.9915, *p* < 0.0001) and Y = 0.9737X + 2.213 (R^2^ = 0.9881, *p* < 0.0001), respectively, suggesting that QDNBs-LFA and FMs-LFA can compete with current commercial quantification methods.

A commercial ELISA test kit was used to analyze a total of 120 clinical medical samples. Figure 7 illustrates the correlation between the TcdB value obtained according to this method and the toxin B value obtained from immunochromatographic analyses. The linear regression equation for QDNBs and FMs were Y = 0.9960X + 0.8703 (R^2^ = 0.9915, *p* < 0.0001) and Y = 0.9737X + 2.213 (R^2^ = 0.9881, *p* < 0.0001), respectively, suggesting that QDNBs-LFA and FMs-LFA can compete with current commercial quantification methods.

## 3. Discussion

QDNBs-LFA and FMs-LFA measurements only require the operator to perform a simple process: mix the excrement samples and functional compounds into the test paper and accurately measure the fluorescent data signal after 12–15 min. These procedures do not require expensive machines/equipment or skilled technicians. Different from traditional medical care methods (e.g., colloidal gold immunochromatographic strips), QDNBs-LFA and FMs-LFA quantification eliminates the subjective distinctions related to human color-depth perception. QDNBs-LFA has distinctive electro-optical characteristics, such as high fluorescent compressive strength, narrow transmission band, large Stokes frequency shift, and long fluorescence lifetime. Moreover, QDNBs-LFA exhibits a lower LOD and shorter field sampling time compared to FMs-LFA. The analysis of the TcdB in the excrement sample indicated similar results between QDNBs-LFA and the commercial ELISA test kit. Therefore, QDNBs are deemed highly suitable for lateral fluidity analyses, which would facilitate the implementation of portable and rapid on-the-spot applications, such as food hygiene and safety tests and onsite medical testing.

## 4. Materials and Methods

### 4.1. Materials and Instruments

TcdA was produced in our laboratory [23]. Human stool samples were supplied by Southern Medical University. Pretrigger and trigger liquids were acquired from Darui Co. Ltd. (Guangzhou, China). Bovine serum albumin (BSA), N-(3-dimethylaminopropyl-N0-ethylcarbodiimide hydrochloride (EDC), N-hydroxysuccinimide (NHS), and AFB1-BSA conjugate were purchased from Sigma-Aldrich (St. Louis, MO, USA). Goat anti-rabbit IgG (secondary antibody) were purchased from Boster Biotech Co. Ltd. (Wuhan, China). QDNBs, which were premodified with polystyrene maleic-anhydride copolymers, were obtained from Shanghai Kundao Biotech Co. Ltd. (Shanghai, China). Fluorescent submicrospheres were obtained from Guangzhou Wondfo Biotech Co. Ltd. (Guangzhou, China), nitrocellulose (NC) membranes, sample pads, absorbent pads, and plastic adhesive cards were purchased from Millipore Co. (Bedford, MA, USA). A commercial human *C. difficile* toxin B (CDT) ELISA kit was supplied by Beijing Huabo Deyi Biotechnology Co., Ltd. (Beijing, China). All other solvents were of analytical grades, and ultrapure water (18.2 MW·cm) was collected from a Millipore Milli-Q system.

TEM images were measured by an FEI electron microscope (Tecnai G2 20 by FEI, Hillsboro, OR, USA). SEM images were acquired with a Zeiss Merlin electron microscope. The photoluminescence of QDNBs and fluorescent submicrospheres was measured with a fluorescence spectrophotometer (Hitachi F-4500, Tokyo, Japan). A dispensing platform (XYZ-3050 by BioDot, Irvine, CA, USA), a guillotine cutter (CM4000 by BioDot, Irvine, CA, USA), and a batch laminator (LM4000 by BioDot, Irvine, CA, USA) were employed to prepare the testing strips. The portable strip reader used herein was acquired from Pureadvance Co., Ltd. (Guangzhou, China). The high-speed freezing centrifuge (CF16RX) was purchased from Hitachi (Tokyo, Japan).

### 4.2. Preparation of TcdB Antigen

The genes coding for the TcdB fragments were amplified from the chromosomal DNA of *C. difficile* VPI10463 (kindly provided by H. Feng, University of Maryland) via polymerase chain reaction (PCR). DNA corresponding to the TcdB fragments (amino acids 1–1851) was amplified from cDNA encoding to full-length TcdB by PCR. The overlapping PCR products and the fragments 1–1851 were cloned into the pHis1525 vector (MoBiTec; Gottingen, Germany) using the BsrGI and KpnI restriction sites. The transformed *E. coli* colonies were then transferred to 100 mL LB broth incorporating 10 µg/mL tetracycline, followed by overnight cultivation at 37 °C and mixing at 250 rpm. This culture was used for recombinant protein isolation via C-terminal His6 tags.

Protein purification was conducted through Ni^2+^ affinity chromatography. Specifically, *B. megaterium* pellets were suspended in 5 mL of 20 mM phosphate sodium buffer (pH = 7.4) containing NaCl (500 mM) and imidazole (30 mM). The sonication-lysed cells were centrifuged at 15,500× *g* and 4 °C for 30 min. The supernatant was passed through a nickel-charged HisTrap HP column (GE Healthcare Bio-Sciences, Pittsburgh, PA, USA). The bound proteins were then eluted using 20 mM phosphate buffer (pH = 7.4) containing NaCl (500 mM) and imidazole (500 mM). Finally, the proteins were dialyzed into 20% glycerol PBS buffer and maintained at −80 °C [24].

### 4.3. Preparation and Identification of Anti-Toxin B Monoclonal Antibodies

Six eight-week-old BALB/c female mice, obtained from the Experimental Animal Center of Nanjing Medical University (Nanjing, China), were acclimated for one week prior to immunization. They were then injected subcutaneously with 100 µg of immunogen emulsified with an equivalent volume of Freund’s complete adjuvant (FCA) for the initial immunization. Afterward, booster immunizations with 50 µg of immunogen, emulsified with an equal volume of Freund’s incomplete adjuvant (FIA), were administered every two weeks. Seven days after the second and third treatments, antibody titers were assessed with ELISA using serum samples obtained from the tail vein. Hybridoma development and identification of monoclonal antibodies were performed as previously described [25]. Antibody affinity was also assessed according to a protocol supplied by the manufacturer [25]. 

### 4.4. Preparation of Signal Immunoconjugates

QDNBs–mAb conjugates were prepared by following previously published QDNBs antibody conjugation protocol [26]. Briefly, 3 mL of QDNBs solution was dispersed in 0.05 M MES (2-[N-morpholino]ethanesulfonicacid, pH 6.0) and activated with 12 mg of EDC and 18 mg of NHS at room temperature for 15 min. Then, the COOH-activated QDNBs were centrifuged at 13,300 rpm for 10 min, and the precipitates were resuspended in 3 mL of borate buffer (0.01 M, pH = 7.4), with gentle agitation for 4 h at room temperature to allow them to react with the antibodies and facilitate the generation of QDNBs–mAb conjugates. Afterward, the mixture was centrifugated again at 13,300 rpm for another 10 min. The obtained precipitates were solubilized in 5 mL of borate buffer (0.01 M, pH = 7.4) containing 1% BSA and gently agitated for an additional 1 h to block the unsaturated sites. Finally, the products were stored at 4 °C.

Polystyrenemicrosphere–antibody (FMs–Ab) signal probes were prepared via the active ether method. First, 8.3 μL EDC solution (1 mg·mL^−1^), 10 μL NHS solution (1 mg·mL^−1^), and 1 mg FMs were added into a 2 mL tube with buffer (0.05 mol·L^−1^ 2-(N-morpholino) and ethanesulfonic acid; MES, pH = 6), followed by shaking at room temperature for 15 min. After centrifuging at 20,817 rpm for 15 min at 4 °C, the precipitate was redissolved in phosphate-buffered saline (PBS; 0.01 mol·L^−1^, pH = 7.4), followed by the immediate addition of 24 μL Ab (1 mg·mL^−1^). The mixture was then shaken at room temperature for 1 h. Afterward, 20 μL of 20% aqueous BSA solution (*w/v*) was added, and the mixture was shaken again at room temperature for another 10 min. The samples were then centrifuged at 20,817 rpm for 15 min at 4 °C. Finally, the precipitate was resuspended in 200 μL of working buffer and stored at 4 °C [27].

### 4.5. Fabrication of the Immunochromatographic Strips

An immunochromatographic strip consists of a sample application pad, nitrocellulose membrane, absorption pad, and a backing card. The anti-TcdB and goat anti-rabbit IgGs that were used for the T line and the C line were coated onto the nitrocellulose membrane using the BioDot XYZ3050 Platform at an adequate jetting rate. The sample pads were then treated with blocking buffers (0.01 M PBS, containing 0.5% (*w/v*) BSA, 0.5% (*v/v*) Tween-20, 0.5% (*w/v*) PVP-K30, 0.25% (*w/v*) EDTA, and 0.05% (*w/v*) sodium azide) and dried overnight at 37 °C. The absorption pad was utilized without treatment. The coated membrane, sample pad, and absorbent pad were laminated and pasted to a plastic scaleboard. After that, the assembled scaleboard was cut laterally with a guillotine cutter (CM 4000) and divided into 4 × 60 mm strips. Finally, the strips were stored in a self-sealing plastic bag with desiccants at 4 °C.

### 4.6. Determination of Immunochromatographic Strip

To enhance the sensitivity and reliability of the testing strips, immunochromatographic strips were optimized, as described previously. Specifically, the pH value, amount of mAb labeled on the QDNBs, amount of QDNBs, amount of mAb labeled on the FMs, amount of FMs, sample volume, and reaction time were optimized. For the assay, the sample was mixed with mAb conjugates using running buffer (distilled water containing 1% sucrose and 1% PVP-K30) in a 96-well plate (the final volume was 100 µL) in duplicate (N = 5). The mixture was loaded onto the sample pad, allowing all liquids to be absorbed and migrated along the strip. After 10 min, the fluorescence intensities of the T line and the C line on the lateral flow test strip were measured with a fluorescence detector at emission wavelengths of 600 and 525 nm, respectively. Quantitative analysis was performed by recording the fluorescence intensities of the T line and the C line, as well as the T/C ratio.

### 4.7. Evaluation of Immunochromatographic Strips

Standard curves of the T/C ratio, as a function of the TcdB concentration, were generated. A TcdB dilution series was prepared by spiking the TcdB standard solution in negative stool samples at 5, 10, 15, 30, 60, 90, and 120 ng/mL, respectively. Each concentration was analyzed in triplicate. To evaluate the standard curves and LOD of immunochromatographic strips, the fluorescence intensities of the T and C lines were used to ensure sensitive and quantitative detection. TcdB recovery was conducted via standard procedures using a series of Tcd-B-spiked samples. Specificity was determined via appropriate amounts of other toxins by cross-reactions of the immunochromatographic strips with TcdA and BSA. Inter- and intrabatch precision was studied using immunochromatographic strips coupled with Tcd-B-spiked samples, and the assay was repeated five times.

### 4.8. Ethics Approval and Consent to Participate

All animal experiments and welfare of the animals were performed under ethical approval from and in agreement with the guidelines of the Guangzhou Wondfo Biotech Co. Experimental Animal Ethics Committee and also in accordance with the policy of the National Ministry of Health. The project identification code is No.43004700043286; the date of approval was 23 March 2018.

## Figures and Tables

**Table 1:** Analytical recovery of TcdB by QDNBs-LFA and FMs-LFA.

Samples (ng/mL)	TcdB (ng/mL) Sample Analyzed by QDNBs-LFA			Samples (ng/mL)	TcdB (ng/mL) Sample Analyzed by FM-LFA		
	Spiked TcdB	Mean Measured Concentration ^1^	Mean Recovery (%)		Spiked TcdB	Mean Measured Concentration ^1^	Mean Recovery (%)
5	5	11.25	112.5	5	5	10.73	107.3
	30	34.57	98.77		30	36.47	104.2
	80	87.3	102.71		80	88.14	103.69
10	5	16.19	107.93	10	5	14.39	95.93
	30	38.23	95.58		30	41.13	102.82
	80	92.56	102.84		80	93.21	103.56
20	5	24.19	96.76	20	5	23.15	92.6
	30	52.41	104.82		30	48.21	98.3
	80	102.84	102.84		80	99.81	99.81

^1^ Mean value of five replicates at each spiked concentration.

**Table 2:** Intra-assay and interassay tests.

LFAs	Samples (ng/mL)	Intrabatch		Interbatch ^1^	
		Mean ^2^	CV%	Mean	CV%
QDNBs-LFA	20	7.9	19.84	6.53	8.81
	40	8.63	41.72	7.41	8.73
	80	6.31	81.14	9.73	9.27
FMs-LFA	20	19.83	3.41	18.75	8.81
	40	37.94	4.31	38.97	8.73
	80	77.47	4.73	83.12	9.27

^1^ Interday assay was completed every day for three consecutive days, with three replicates for each concentration^2^. Mean value of five replicates for each spiked concentration.
